# Clinical Application of Transcutaneous Microcurrent Therapy for an Acute Traumatic Contusion in a Special Operations Military Officer: A Case Report

**DOI:** 10.3390/life16081370

**Published:** 2026-08-20

**Authors:** Ignacio Aparicio-Arranz, David Martín-Caro Álvarez, Emilio Rodríguez-Ramos, Ana Onate-Figuérez, Juan R. Godoy-López, Juan Avendaño-Coy

**Affiliations:** 1 Faculty of Biomedical and Health Sciences, Alfonso X el Sabio University, 28691 Madrid, Spain; nachoa.pf2@gmail.com; 2 Toledo Physiotherapy Research Group (GIFTO), Faculty of Physiotherapy and Nursing, University of Castilla-La Mancha, 45071 Toledo, Spain; ana.onate@uclm.es (A.O.-F.); juan.avendano@uclm.es (J.A.-C.); 3 Toledo Physiotherapy Research Group (GIFTO), Castilla-La Mancha Health Research Institute (IDISCAM), 45004 Toledo, Spain; 4 Training Department, Central School of Physical Education of the Spanish Army (ECEF), 45009 Toledo, Spain; emilioramos03@hotmail.com (E.R.-R.); jgodoylo@et.mde.es (J.R.G.-L.)

**Keywords:** microcurrent, creatine kinase, special forces, traumatic contusion, foot

## Abstract

Contusions are highly prevalent injuries in the armed forces. They can substantially impair physical performance, particularly in special operations forces, where operational readiness depends on maintaining a high level of physical fitness. Transcutaneous microcurrent therapy (TMCT) is a non-invasive intervention that has been reported to promote tissue repair. The aim of this case report was to describe the evolution of creatine kinase levels, functional outcomes, pain, and perceived well-being during TMCT following an acute traumatic contusion. Case presentation: A 34-year-old special operations military officer with an acute traumatic contusion in the dorsum of the left foot received TMCT applied over the injured area for 72 h. The device delivered a monophasic rectangular current at 21 mV and 42 μA, with a total application time of 2360 min over the 72-h observation period. CK and the Hooper Index were assessed at baseline and at 24, 48, and 72 h, whereas NPRS and LLFI were assessed at baseline and at 72 h. Over the 72-h observation period, CK decreased from 571 to 203 IU/L, the Hooper Index decreased from 15 to 12 points, the NPRS score decreased from 7 to 6 points, and the LLFI score increased from 52% to 84%. No intervention-related adverse events were reported. Conclusions: During the 72-h observation period, reductions in CK and pain and an increase in perceived lower-limb function were observed while the participant was receiving TMCT. These preliminary observations support further investigation of TMCT as a potential adjunct to standard conservative care. However, the single-case design and the absence of a control condition prevent the observed changes from being attributed to TMCT. Controlled studies are required to evaluate its efficacy and clinical applicability.

## 1. Introduction

Lower-extremity and foot injuries represent a substantial health burden among military personnel exposed to high physical demands. Approximately one in four US active-duty service members sustained a lower-extremity musculoskeletal injury in 2021, while 7.5% sustained a foot or toe injury [1]. Furthermore, a systematic review including 8,092,281 military personnel recorded 788,469 foot and ankle injuries, with acute injuries being considerably more frequent than non-acute injuries [2]. However, the specific epidemiology of acute traumatic contusions affecting the dorsum of the foot remains poorly characterized. Direct trauma to this area may cause soft-tissue damage and should be differentiated from a true Lisfranc injury involving ligamentous and/or osseous damage to the tarsometatarsal complex. The Lisfranc region plays an important role in load transmission and foot stability, and injuries affecting this complex may result in considerable functional impairment [3]. In the present manuscript, the term “Lisfranc region” is used only to describe the anatomical location of the dorsal-foot contusion and does not imply a confirmed ligamentous, osseous, or unstable Lisfranc injury. When a true Lisfranc injury is confirmed, management depends on joint stability: stable injuries may be treated conservatively with immobilization and restricted weight-bearing, whereas unstable injuries generally require surgical treatment [3,4]. These management strategies are therefore provided as differential-diagnostic context and are not directly applicable to the soft-tissue contusion described in this case.

Transcutaneous microcurrent therapy (TMCT) is a non-invasive electrotherapeutic modality that delivers low-intensity subsensory electrical currents in the microampere range [5]. Clinical studies evaluating portable TMCT devices have reported a low incidence of treatment-related adverse events [6] and have shown that prolonged applications can be feasible and well tolerated [7]. At the experimental cellular level, microcurrents have been reported to increase ATP production, stimulate protein synthesis, and enhance amino acid transport, thereby supporting anabolic and bioenergetic processes [8]. In contrast, clinical evidence related to tissue repair has mainly been obtained in chronic human skin wounds, in which shorter healing times have been reported [7]. Additional experimental studies have suggested that TMCT may support skeletal muscle regeneration through increased satellite-cell activation [9].

To the best of our knowledge, direct clinical evidence regarding the application of TMCT in acute traumatic dorsal-foot contusions remains very limited. Therefore, this case report provides a descriptive account of the temporal evolution of biochemical, clinical, and functional outcomes during TMCT application, together with treatment tolerability and intervention-related adverse events.

The primary objective of this case report was to describe the temporal evolution of creatine kinase levels during the application of TMCT in a patient with an acute traumatic contusion. The secondary objectives were to describe the observed changes in pain perception, lower-limb function, and perceived well-being, as well as to record any intervention-related adverse events.

## 2. Materials and Methods

The present study was approved by the local Ethics Committee of Toledo (ref. no. 2364; 25 March 2026). The participant was informed about the study protocol and signed the informed consent to participate in the study and for the publication of his clinical data and case-related images. This study was conducted at the Central School of Physical Education, located within the Infantry Academy in Toledo, Spain. Founded in 1919, this century-old Spanish Army institution is devoted to education and specialization in physical education and the physical training of military personnel.

### 2.1. Case Presentation

A 34-year-old special operations officer, 175 cm tall and weighing 87 kg, with no relevant medical history, sustained an acute traumatic contusion to the dorsum of the left foot during a close-quarters combat training exercise 48-h before the initiation of TMCT. Clinical examination revealed a contusion of the dorsal soft tissues overlying the Lisfranc region. No specific Lisfranc ligament, dorsal foot muscle, or structural lesion such as a partial tear or deep hematoma could be identified from the available clinical data, and a complete imaging-based diagnosis was not available. The injury caused local swelling, tenderness to palpation, a pain intensity of 7/10 on the NPRS and significant functional impairment. Before TMCT application, the participant was screened for potential contraindications, including the presence of implanted electronic devices, pregnancy, seizure disorders, and relevant neurological or sensory disorders. None of these contraindications were present. During the 72-h period, the participant did not take anti-inflammatory medication and did not receive physiotherapy, any other therapeutic intervention, or any specific recovery measure. Furthermore, he did not perform any additional activity beyond the duties associated with his instruction period.

### 2.2. Intervention

**Figure 1:**
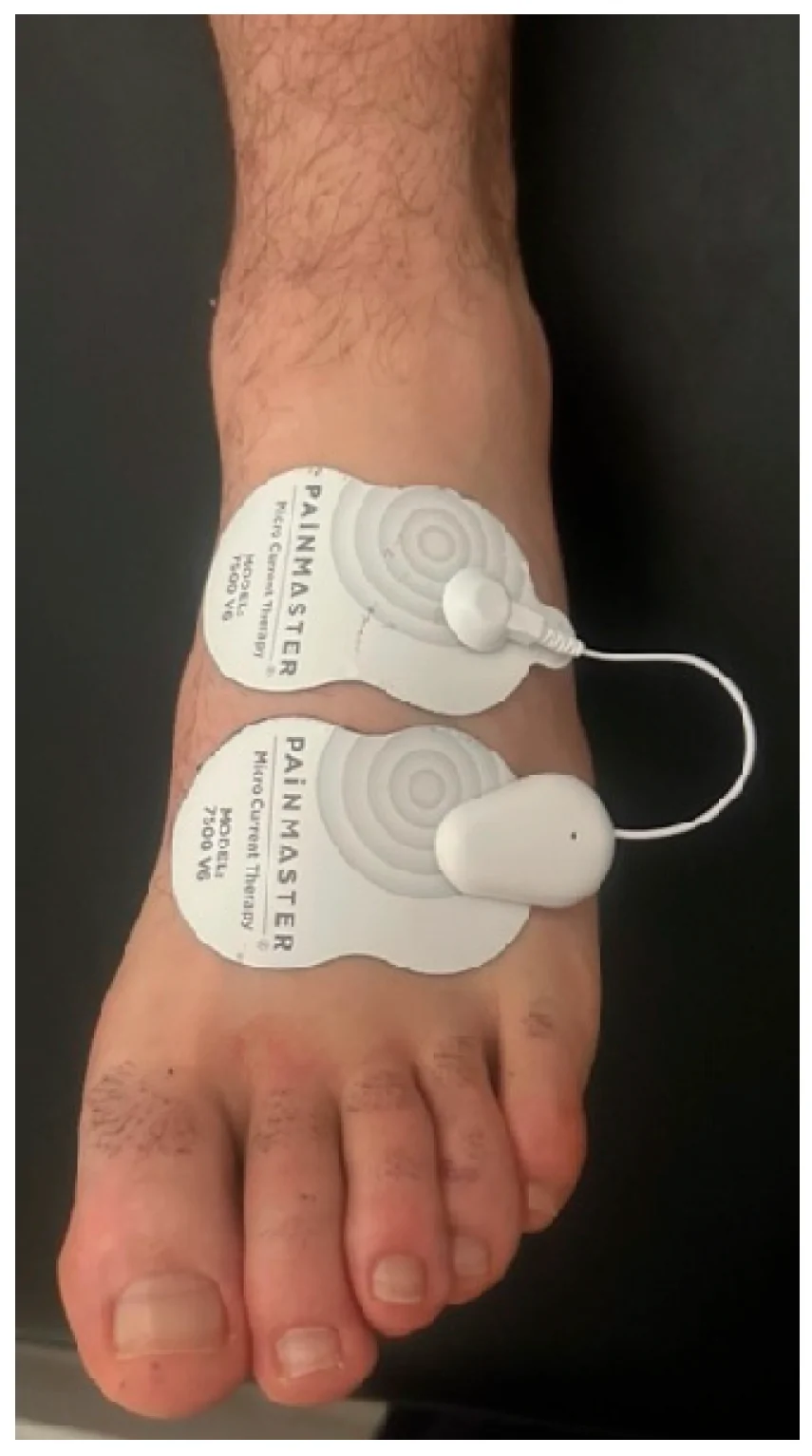
TMCT electrode placement.

TMCT was administered using the MC Patch^®^ device (Newmark, Inc., 131 Quarry Village Rd, Cheshire, CT, USA). Two electrode pads were placed adjacent to each other over the contused area on the dorsum of the foot, with one pad positioned proximally and the other distally along the longitudinal axis of the foot, as shown in Figure 1. The device delivered a monophasic rectangular current at a voltage of 21 mV and a fixed intensity of 42 μA. these parameters correspond to the preset output characteristics of the MC Patch^®^ device and were not individually selected or modified by the investigators. A previous randomized double-blind, sham-controlled clinical trial using the same device applied the same parameters, supporting the feasibility and safety of prolonged subsensory microcurrent application. However, these parameters should not be considered optimal, as dose–response comparisons have not been conducted yet [7]. The application time by the participant was 890 min on the first day, 660 min on the second day, and 810 min on the third day, resulting in a total exposure of 2360 min over the 72-h intervention period. The variation in daily application time was related to the participant’s daily schedule and to temporary device removal during personal hygiene and activities in which the electrodes could not be safely maintained. Outcome assessments were performed at baseline, immediately before device placement, and approximately 24, 48, and 72 h after the start of the intervention. Consequently, each assessment reflected the participant’s cumulative exposure to TMCT up to that time point.

### 2.3. Outcome Measures

The primary outcome measure was creatine kinase (CK), and the secondary outcome measures were Numeric Pain Rating Scale (NPRS), Lower Limb Functional Index (LLFI), and Hooper Index. CK and the Hooper Index were assessed at four time points: baseline and 24, 48, and 72 h after the start of the intervention. NPRS and LLFI were assessed at baseline and at the final 72-h assessment. At the end of the intervention, the participant was asked about device comfort and acceptability, including whether its use had caused any discomfort or interfered with his usual activities.

#### 2.3.1. Creatine Kinase

CK is an intracellular muscle enzyme that catalyzes the reversible reaction between creatine and phosphocreatine, which is essential for the rapid resynthesis of ATP during exertion. This measurement is a valid biomarker for quantifying muscle damage and is considered one of the most reliable physiological measures available. Elevated blood CK levels may indicate disruption of muscle fiber integrity and the release of intracellular contents into the bloodstream [10]. CK analysis following trauma is a sensitive biochemical measure for monitoring muscle damage and soft-tissue involvement and is useful for interpreting the biochemical response after trauma [11].

The participant’s CK levels were analyzed using the Simplex TAS 101^®^ biochemical analyzer (TASCOM Co., Ltd., Anyang-si, Republic of Korea), a point-of-care (POC) diagnostic device validated for diagnostic use in the European Union (CE marked). CK cartridge requires 20 μL of whole blood and has an analytical measurement range of 10–1200 IU/L, with a lower measurement limit of 10 IU/L. Technical comparative data obtained from 428 samples showed a strong linear correlation between CK values measured with the SimplexTAS 101^®^ and those obtained using the Cobas c 311 laboratory analyzer (y = 1.011x − 0.6979; R^2^ = 0.994). These analytical characteristics support the reliability of the device for rapid, serial point-of-care CK measurements. The device determined enzyme activity in 14 min, with results expressed in International Units per liter (IU/L). This primary outcome measure was assessed at four time points: pre-intervention and at 24, 48, and 72 h post-intervention.

#### 2.3.2. Numeric Pain Rating Scale (NPRS)

The Numeric Pain Rating Scale (NPRS) is a subjective patient-reported measure designed to quantify perceived pain intensity. The participant selects an integer on an 11-point scale, from 0, representing no pain, to 10, representing the worst pain imaginable. This scale allows changes in pain intensity to be monitored throughout the recovery process [12] and has demonstrated adequate validity for the assessment of acute pain [13]. Pain intensity was assessed at rest under the same conditions at baseline and at the 72-h evaluation.

#### 2.3.3. Lower Limb Functional Index (LLFI)

The LLFI is a patient-reported outcome measure designed to assess perceived lower-limb function. The questionnaire consists of 25 items scored as 1 (“Yes”), 0.5 (“Partly”), or 0 (“No”). The item scores are summed, multiplied by four, and subtracted from 100 to obtain a final functional score ranging from 0 to 100%, with higher scores indicating better lower-limb function and lower disability [14]. Although the LLFI is not specific to a particular injury, it has demonstrated high validity and reliability for assessing lower-limb function by directly reflecting the patient’s perspective [15].

#### 2.3.4. Hooper Index

The Hooper and Mackinnon questionnaire, or Hooper Index, is a subjective tool for monitoring general well-being. It assesses four daily psychophysiological states: perceived general fatigue, muscle soreness, sleep quality, and stress levels. Each item is scored on a 7-point Likert scale, ranging from 1 (optimal state) to 7 (worst state). The sum of the four items provides an overall score of the participant’s well-being [16].

### 2.4. Data Analysis

Given the single-case design of this study, a longitudinal within-subject descriptive analysis was performed. The primary outcome measure (CK) and the secondary outcome measure (Hooper Index) were assessed at their respective time points after baseline. Other secondary measures—including the Lower Limb Functional Index (LLFI) and the Numeric Pain Rating Scale (NPRS)—were assessed before and after the intervention. To quantify variations, absolute and percentage changes from baseline values were calculated. Standard inferential statistical tests were not applied because the sample consisted of a single participant; instead, data interpretation was based on the temporal evolution and clinical relevance of the findings.

## 3. Results

**Figure 2:**
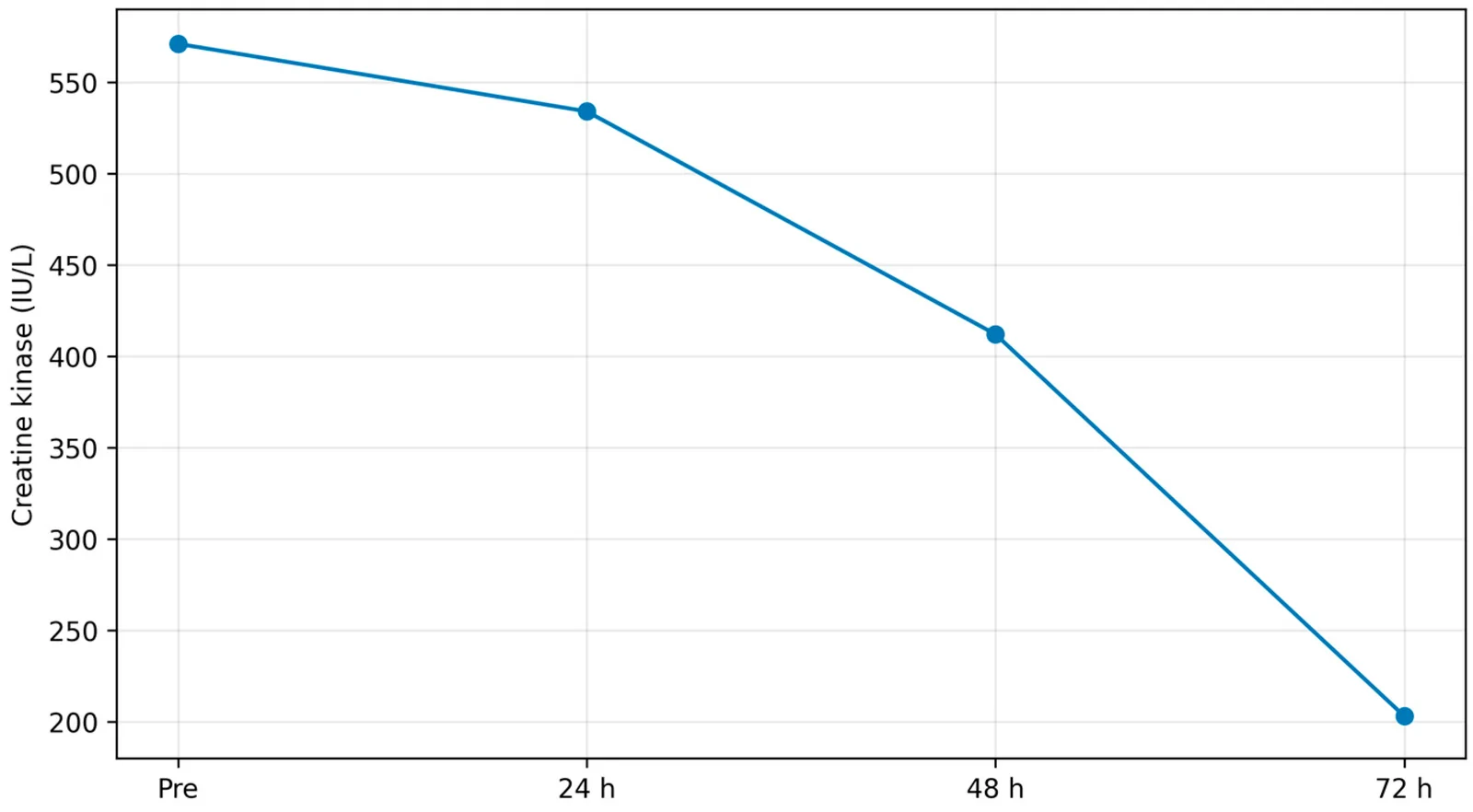
Time course of creatine kinase values (IU/L).

**Figure 3:**
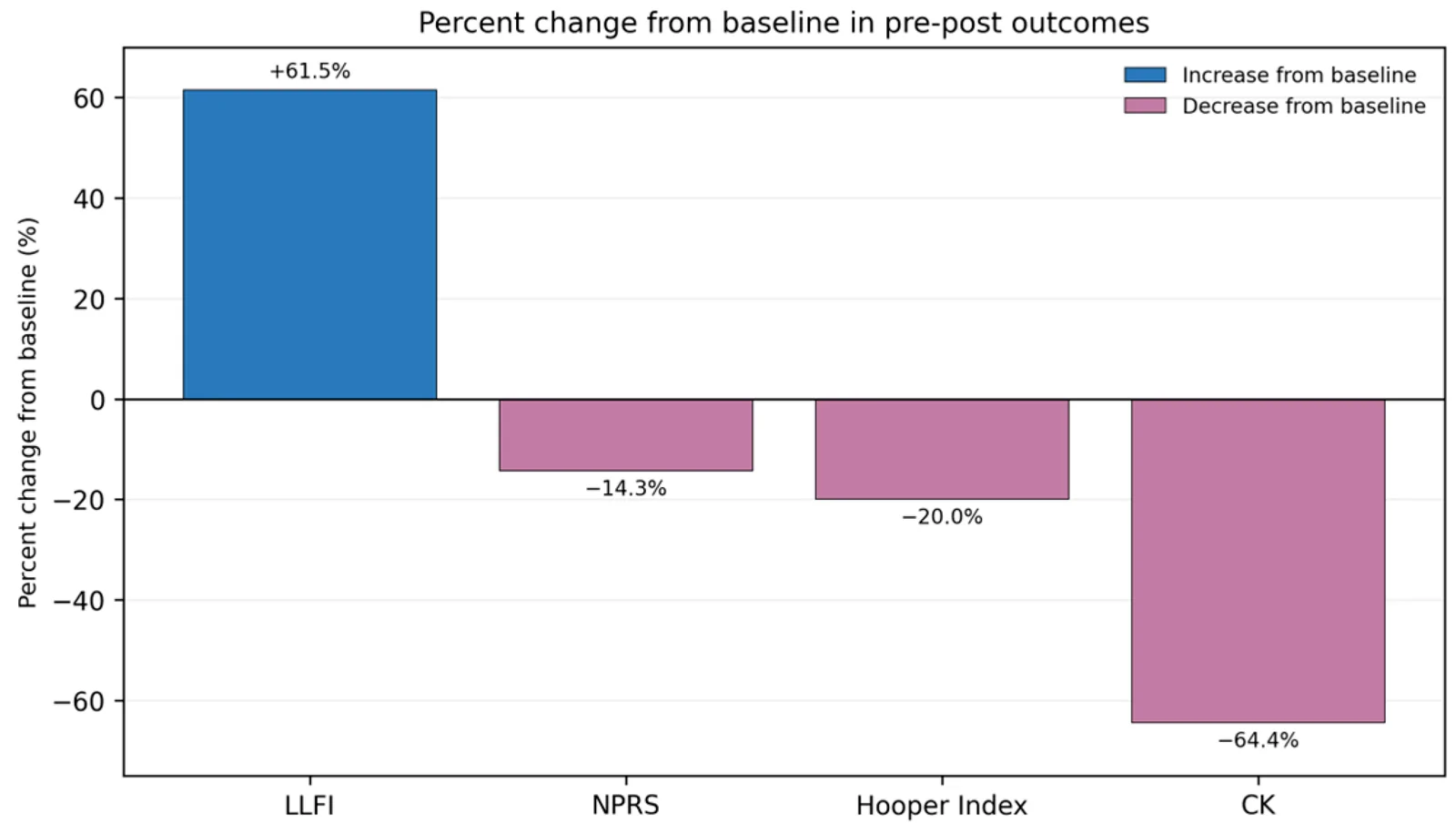
Percentage change from baseline to the final assessment (72 h) across all study outcomes.

Table 1 shows the results obtained for the outcomes of the study throughout the intervention. CK levels decreased from 571 IU/L at baseline to 534 IU/L at 24 h, 412 IU/L at 48 h, and 203 IU/L at 72 h (Figure 2), representing a cumulative reduction of 64.4% compared to baseline. A single physiological reference interval was not applied because CK values are highly dependent on individual characteristics, including sex, muscle mass, physical activity, and training status [10]. The Hooper Index showed a non-linear evolution during the follow-up period, increasing from 15 at baseline to 17 at 24 h and subsequently decreasing to 12 at 48 h, with the same value maintained at 72 h. Regarding outcomes assessed exclusively pre- and post-intervention: LLFI showed a 61.5% improvement in the participant’s function compared to baseline, increasing from 52% to 84% at 72 h (Figure 3). Pain perception using the NPRS decreased from 7 points to 6 points at post-intervention, representing an absolute reduction of 1 point. This change did not reach the 1.3-point minimum clinically significant difference reported for the NPRS in acute pain [13].

**Figure 4:**
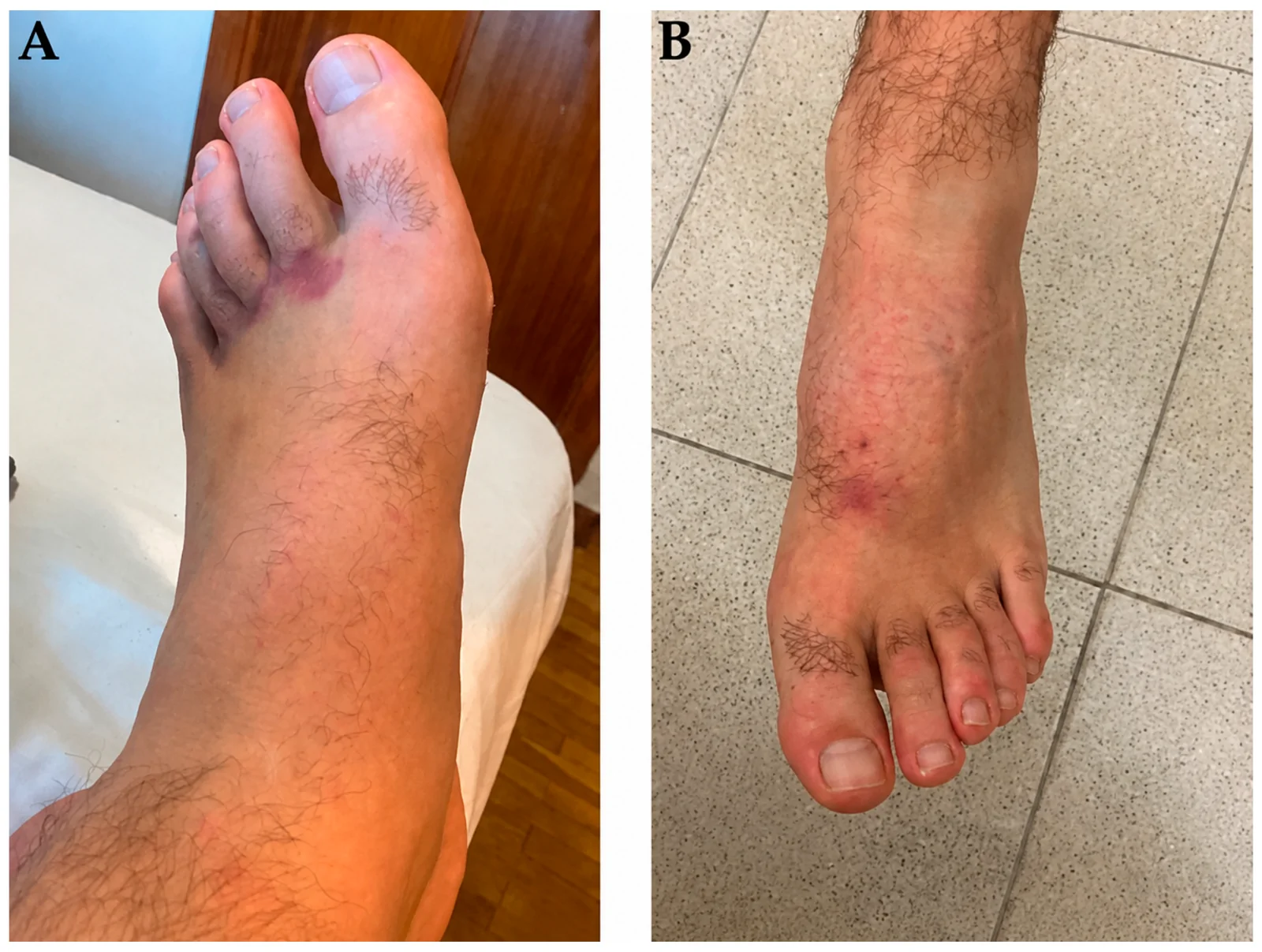
Clinical appearance of the injured foot at baseline and after 72 h of follow-up. (**A**) Pre-intervention, (**B**) post 72 h TMCT.

At baseline, the participant presented marked local swelling over the dorsal aspect of the injured foot. This swelling decreased during the 72-h follow-up period, as illustrated in Figure 4, and an improvement in foot mobility was also clinically observed; however, this change was not objectively quantified. No adverse effects were reported during application.

The participant received a total treatment time of 2360 min via TMCT. This total time was distributed over a 72-h period as detailed in Table 2. The participant met the prescribed minimum application time of 360 min/day on all three intervention days. Participant reported that the device was comfortable, particularly because it was mainly worn at night. He also stated that it caused no discomfort and did not interfere with his usual daytime activities. No skin irritation, unpleasant sensations, or other device-related problems were reported.

## 4. Discussion

After 72 h of intervention, changes were observed in the biochemical, functional, and patient-reported outcomes. CK decreased from 571 to 203 IU/L, the Hooper Index changed from 15 at baseline to 17 at 24 h and then decreased to 12 at 48 and 72 h, NPRS decreased from 7 to 6 points, and LLFI increased from 52% to 84%. NPRS and LLFI were predefined as pre–post measures, whereas CK and the Hooper Index were monitored serially at baseline and at 24, 48, and 72 h. Therefore, the intermediate course of pain and perceived lower-limb function at 24 and 48 h cannot be determined. Previous clinical and experimental studies provide indirect biological support for TMCT in other contexts; however, they do not constitute direct evidence for acute traumatic dorsal-foot contusions [7,17]. Daily TMCT exposure varied across the three intervention days (890, 660, and 810 min). Although CK decreased throughout follow-up, the single-case design, limited number of measurements, and expected physiological clearance of CK prevent determining whether variations in daily exposure influenced CK kinetics or clinical recovery. Accordingly, no dose–response relationship can be inferred, and future controlled studies should use a more standardized treatment dose.

The observed reduction in CK should be interpreted with caution, as this marker may naturally decrease in the hours following an acute muscle injury and therefore cannot be attributed exclusively to the intervention [18]. CK concentrations may decrease as part of the natural biological recovery process following an acute traumatic injury, independently of the treatment applied. Furthermore, CK responses show considerable interindividual variability and may be influenced by other factors [10]. The same applies to the Hooper Index; although it is not an injury-specific scale, it captures relevant information on the patient’s general condition [19]. The observed changes in CK, the Hooper Index, LLFI, and NPRS reflect different aspects of the participant’s course during follow-up. However, these outcomes assess distinct dimensions and were not all evaluated at the same time points; therefore, their temporal changes should not be interpreted as directly parallel or mutually confirmatory. Taken together, these findings suggest that TMCT could be a useful adjunctive tool within the conservative management of acute traumatic contusions.

The soft-tissue contusion described in this case should be distinguished from a true Lisfranc injury involving ligamentous damage, joint instability, or fracture. In confirmed Lisfranc injuries, the indication for immobilization and weight-bearing restriction depends on the specific diagnosis and joint stability [4]. However, such interventions are associated with disuse muscle atrophy, joint stiffness, reduced tissue elasticity, and extracellular matrix alterations, including increased collagen deposition and muscle fibrosis [20,21]. In joints exposed to high mechanical demands, such as the Lisfranc region, these adverse effects may be even more pronounced, leading to increased joint stiffness and reduced range of motion [22,23]. Although causal conclusions cannot be drawn from a single case, these findings support further investigation of TMCT as a potential adjunctive approach in the conservative management of acute traumatic contusions. From a military perspective, interventions that accelerate functional recovery could help reduce healthcare costs and minimize the impact of musculoskeletal injuries on operational readiness, as injured service members limit unit deployment capability and increase healthcare resource utilization [24].

Previous studies have reported favorable effects of microcurrent therapy on CK, muscle function, pain, and tissue healing. However, these studies involved exercise-induced muscle damage, chronic wounds, musculoskeletal pain, or experimental models, with different populations and treatment protocols. Therefore, their findings cannot be directly extrapolated to an acute traumatic foot contusion. Specific clinical evidence for TMCT in this condition remains limited, and the present findings should be interpreted as preliminary and hypothesis-generating.

### 4.1. Practical Applications

TMCT may represent an adjunctive strategy for managing traumatic contusions, particularly in athletes and military personnel, for whom physical availability is essential to maintaining performance. Special operations soldiers, often referred to as tactical athletes [25], are exposed to high physical demands under stressful and challenging environmental conditions. Consequently, traumatic foot injuries can substantially impair operational performance and mission readiness. In this context, the compact and portable nature of the device and the absence of an external power source may facilitate its use in military or sports settings. However, the prolonged application time observed in this case (approximately 11–15 h/day) may limit adherence and its practical applicability in routine clinical or military activity.

This single case does not allow identification of the patients most likely to benefit from TMCT. However, its potential use may be considered as an adjunct to conservative management in physically active individuals with acute traumatic soft-tissue contusions, following appropriate clinical assessment and exclusion of injuries requiring specific management. TMCT should therefore be considered as an adjunct rather than a replacement for standard care.

### 4.2. Study Limitations and Future Research

The inherent limitations of a case report, including the absence of a control group and possible subjective bias in some patient-reported outcome measures, restrict the generalizability of the findings. In addition, the short follow-up period prevents evaluation of the long-term effects of the intervention. A complete imaging-based diagnosis was not available, and standardized objective criteria were not used to classify the severity of the contusion. Daily TMCT application time varied across the three intervention days. Furthermore, CK concentrations may change as part of the natural biological course following acute trauma and may also be influenced by physical activity. Although the absence of anti-inflammatory medication, physiotherapy, other therapeutic interventions, and additional activity during follow-up was documented, the influence of potential confounding factors cannot be completely excluded. Finally, several outcomes were patient-reported, and some clinical changes, such as foot mobility, were not objectively quantified using standardized measures.

Future research should initially include a randomized, double-blind, sham-controlled pilot clinical trial involving 50 participants, with 25 participants allocated to each group. Participants should be randomly assigned in a 1:1 ratio to active TMCT or to a visually identical sham device, while both groups receive the same standardized conservative care recommendations. Allocation concealment should be implemented, and participants and outcome assessors should remain blinded to group assignment.

The change in CK concentration from baseline to 72 h could be established as the primary outcome. Secondary outcomes should include NPRS, LLFI, the Hooper Index, objective clinical findings, treatment tolerability, adverse events, and time to full return to physical or occupational activity. Assessments should be conducted at baseline and at 24, 48, and 72 h, with additional follow-up assessments at 7, 14, and 28 days. The pilot trial would provide information on recruitment, adherence, retention, outcome variability, and the feasibility of blinding, while enabling an a priori sample-size calculation for a subsequent adequately powered definitive randomized controlled trial.

## 5. Conclusions

In this case report, reductions in CK and pain and improvements in lower-limb function and perceived well-being were observed during 72 h of TMCT application, with no intervention-related adverse events reported. Nevertheless, the single-case design, the absence of a control condition, the natural course of acute contusions, and potential confounding factors prevent these changes from being attributed to TMCT. In high-demand military and tactical populations, the portable and well-tolerated nature of TMCT may support its potential use as an adjunct to standard conservative care, although its clinical efficacy remains to be established. These preliminary observations may contribute to the design of future randomized, sham-controlled studies evaluating TMCT as an adjunct to standard care.

## Figures and Tables

**Table 1:** Results of the outcome measures assessed in the study. IU/L: International Units/liter.

Results	Pre-Intervention	24 h	48 h	72 h
CK (IU/L)	571	534	412	203
NPRS(points)	7	-	-	6
LLFI (%)	52	-	-	84
Hooper Index (points)	15	17	12	12

**Table 2:** Daily TMCT application time (min).

Application Date	Application Time TMCT (min)
15 June 2026	890
16 June 2026	660
17 June 2026	810
Total time	2360

## Data Availability

All data are included in the manuscript.

## Abbreviations

TMCT	Transcutaneous microcurrent therapy
CK	Creatine kinase
LLFI	Lower Limb Functional Index
NPRS	Numeric Pain Rating Scale
POC	Point of care
